# The relationship between remote diffusion-weighted imaging lesions and the triglyceride-glucose index and clinical outcomes in patients with intracerebral hemorrhage

**DOI:** 10.3389/fneur.2025.1562361

**Published:** 2025-07-21

**Authors:** Lu Wang, Yu Gu, Guoliang Jiang, Chunyan Lei, Potao Zhang, Wen Jiang, Xinglong Yang, Ansong Jin, Qionghua Deng

**Affiliations:** ^1^Department of Neurology, The First Affiliated Hospital of Kunming Medical University, Kunming, Yunnan, China; ^2^Department of Interventional Medicine, The First Affiliated Hospital of Kunming Medical University, Kunming, Yunnan, China

**Keywords:** intracerebral hemorrhage, DWI lesions, triglyceride-glucose index, clinical outcome, insulin resistance

## Abstract

**Objective:**

This study aimed to observe the relationship between the presence of distal diffusion-weighted imaging (DWI) lesions and triglyceride-glucose (TyG) index and clinical outcome after intracerebral hemorrhage (ICH), and identify the risk factors for DWI lesions in ICH patients.

**Methods:**

ICH patients at the First Affiliated Hospital of Kunming Medical University were retrospectively collected. Demographic data, laboratory examination, and imaging data of the patients were collected. The patients were divided into two groups based on the presence or absence of distal DWI lesions as determined by magnetic resonance imaging (MRI). Multivariate logistic regression analysis was used to evaluate the risk factors for DWI lesions and clinical outcomes.

**Results:**

Among 245 ICH patients included in this study, 46 (18.78%) had DWI lesions and 199 (81.22%) did not. We found that the occurrence probability of DWI lesions reached the maximum in the range Q2 of the TyG index. ICH patients with DWI lesions had a similar frequency of death or disability at 90 days compared with patients without DWI lesions. Multivariate logistic regression analysis showed that high fasting glucose (*p* = 0.039) and hematoma site (*p* = 0.048) were significant predictors of DWI lesions after ICH. The old age (*p* < 0.001), higher National Institutes of Health Stroke Scale (NIHSS) score (*p* < 0.001), and midline shift (*p* = 0.034) were independent predictors of poor functional outcome at 3 months.

**Conclusion:**

There was no definitive correlation between the TyG index and distal DWI lesions in our study. The elevated high fasting glucose levels and hematoma site were significant predictors for DWI lesions after ICH.

## Introduction

Intracerebral hemorrhage (ICH) is the second most common subtype of stroke after ischemic stroke. Only about 20% of patients can recover their self-care ability after 6 months, which brings a heavy burden to society and family (1). The outcome of patients with ICH is related to clinical severity and hematoma volume (2, 3). In recent years, with the application of magnetic resonance imaging (MRI), distal diffusion-weighted imaging (DWI) lesions have been found in patients with ICH. It is currently believed that these DWI lesions represent active ischemia. Relevant studies have suggested that these lesions were associated with poor clinical outcomes in patients with ICH (1, 3–8). At present, the influencing factors and potential mechanisms behind these findings remain unclear (4, 5, 9).

Insulin resistance (IR) is a prominent characteristic of metabolic disorders, and the hyperinsulin–normal glucose clamp test (HEC) is considered the gold standard for evaluating insulin resistance. However, the clinical application of HEC is severely limited due to the complex testing process, lengthy duration, and high cost (10). Previous studies have shown that the fasting triglyceride-glucose (TyG) index was convenient, rapid, cost-effective, reliable, and an alternative indicator of insulin resistance. TyG has shown significant correlation with HEC and homeostatic model assessment of insulin resistance (HOMA-IR), suggesting its potential use as a substitute indicator (11, 12).

Previous studies have shown that there was a known relationship between the TyG index and ischemic stroke, with a higher TyG index being linked to an increased risk of ischemic stroke (13). However, it remains unclear whether the TyG index is correlated with DWI lesions following ICH, and the evidence regarding DWI lesions and clinical outcomes in ICH patients is also limited (3, 14, 15). This study aimed to investigate the relationship between the TyG index and DWI lesions in patients with ICH, identify the risk factors for DWI lesions after ICH, and examine the association between DWI lesions and clinical outcomes in patients with ICH.

## Subjects and methods

This study retrospectively included patients with ICH admitted to the First Affiliated Hospital of Kunming Medical University from July 2019 to July 2024. The study protocol was designed in accordance with international ethical guidelines for human research and was approved by the Scientific Research Department of the First Affiliated Hospital of Kunming Medical University [Ethics number: (2024) Ethics L No. 189].

The inclusion criteria were as follows: (1) age ≥18 years, (2) diagnosis of spontaneous intracerebral hemorrhage, not limited to the first intracerebral hemorrhage, and (3) a head MRI performed within 1 month. The exclusion criteria were as follows: (1) Traumatic cerebral hemorrhage, (2) secondary cerebral hemorrhage due to aneurysm, vascular malformation, moyamoya disease, cavernous hemangioma, cerebral venous thrombosis, tumor, or hemorrhagic transformation of cerebral infarction, (3) patients taking on long-term anticoagulant drugs for a long time, and (4) patients with incomplete data.

### Baseline data and etiological classification

Baseline information was collected for each enrolled patient, including (1) demographic data such as sex and age; (2) clinical characteristics such as time from onset of symptoms to admission, time from onset to magnetic resonance imaging (MRI), admission status [vital signs, clinical manifestations, consciousness, Glasgow Coma Scale (GCS) at admission and National Institutes of Health Stroke Scale (NIHSS)], previous medical history (hypertension, diabetes, hyperlipidemia, ischemic stroke, and coronary heart disease), type of medication taken in the past (including antiplatelet drugs, antihypertensive drugs, lipid-lowering drugs, hypoglycemic drugs, etc.), personal history (smoking, drinking, etc.), and family history; (3) laboratory examination, which involved measuring basic biological indicators (blood routine, liver and kidney function, blood glucose, blood lipids, glycated hemoglobin, coagulation, etc.); (4) treatment options, including conservative treatment and surgical treatment.

The TyG index was calculated according to the collected fasting blood glucose and blood lipid data. The TyG index can be calculated as follows: ln (fasting triglyceride (mg/dL) × fasting glucose (mg/dL)/2) (16–18).

### Image features

According to the protocol, imaging data should be collected for each enrolled ICH patient, including head CT on admission, to assess the hematoma location and volume (19), presence of subarachnoid hemorrhage, and intraventricular hemorrhage in the patient. Hematoma volume was determined using the ABC/2 formula (20).

MRI was performed with a 3.0-Tesla scanner within 1 month of symptom onset. The sequences comprised axial T1-weighted, T2-weighted, T2-fluid-attenuated inversion recovery (FLAIR), DWI, and apparent diffusion coefficient (ADC). The collected data included DWI lesions, white matter hyperintensities (WMH), enlarged perivascular spaces (EPVS), lacunar infarction, and brain atrophy.

**Figure 1:**
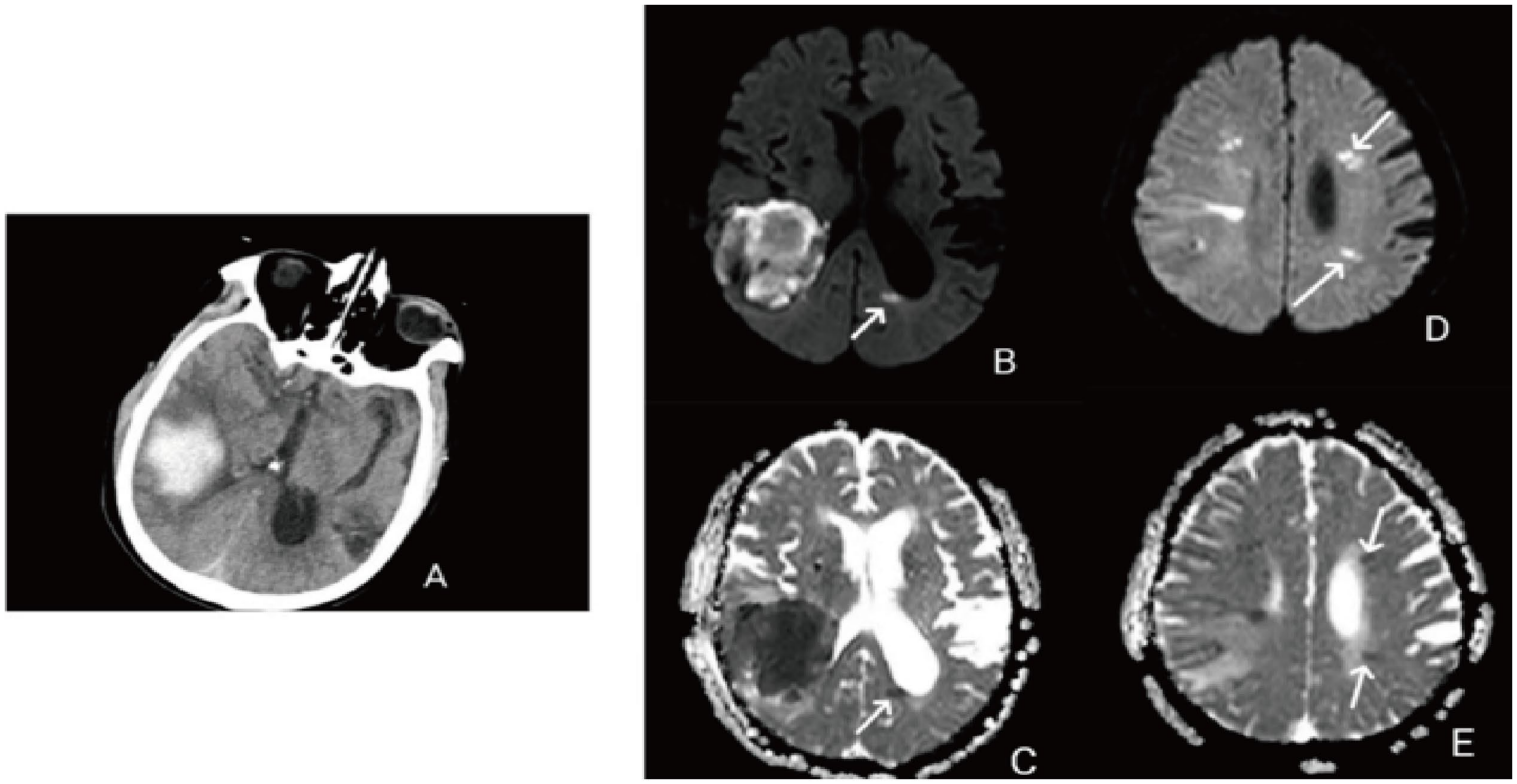
**(A)** Right temporal lobe hemorrhage; **(B)** remote diffusion-weighted imaging lesions (R-DWILs) in posterior horn of the left lateral ventricle; **(C)** low signal on the ADC corresponding to the posterior horn of the left lateral ventricle; **(D)** remote diffusion-weighted imaging lesions (R-DWILs) in the left frontal lobe and parietal lobe; **(E)** low signal on the ADC corresponding to the left frontal lobe and temporal lobe.

Remote diffusion-weighted imaging (R-DWI) lesion is defined as a focal high signal on DWI and a corresponding low signal on the apparent diffusion coefficient (ADC), excluding limited diffusion within or adjacent to the hematoma (<20 mm) (Figure 1) (3, 21).

WMH includes periventricular white matter (PVWM) and deep white matter (DWM). The Fazekas scale was used to evaluate the presence and extent of WMH. The values of PVWM and DWM range from 0 to 3. The total score on the Fazekas scale is obtained by summing the scores of PVWM and DWM. Specifically, a score of 0 is classified as Fazekas 0, a score of 0–2 is classified as Fazekas 1, a score of 3–4 is classified as Fazekas 2, and a score of 5–6 is classified as Fazekas 3 (22, 23).

EPVS were defined as small, clearly depicted structures of cerebrospinal fluid intensity on imaging that followed the orientation of the perforating vessels and ran perpendicular to the brain surface. They exhibited high signal on T2 and low signal on T1 and fluid-attenuated inversion recovery sequences. The number of EPVS in the basal ganglia and center of the semi-oval EPV was categorized as follows: 0 = no EPVS, 1 ≤ 10 EPVS, 2 = 10–20 EPVS, 3 = 21–40 EPVS, and 4 ≥ 40 EPVS. Midbrain EPVS were classified as visible or invisible (22, 24, 25).

All imaging data were assessed by two experienced physicians who were blinded to the patient’s clinical information prior to evaluation. In cases of disagreement between the two reviewers, the third reviewer would assess the data and finally reach an agreement (*κ* = 0.95).

### Clinical outcomes

Primary outcome: relationship between the TyG index and distal DWI lesions. Secondary clinical outcome: distal DWI lesions and clinical outcome. Patient follow-up was conducted via telephone at 90 days after stroke onset. Experienced neurologists, who were blinded to patient data, calculated modified Rankin Scale (mRS) scores and mortality of each patient. A good functional outcome was defined as an mRS score of 0–2, while a poor functional outcome was defined as an mRS score of 3–6.

### Statistical analysis

Data were analyzed using Statistical Package for Social Sciences (SPSS) version 26.0 software (IBM Corporation, Armonk, NY, United States). Normality test using the Kolmogorov–Smirnov test and the Shapiro–Wilk test was carried out to assess all quantitative variables. Some quantitative variables that conformed to a normal distribution were expressed as mean ± standard deviation (SD), and an independent-sample *t*-test was used to compare the two groups. The other data, not conforming to a normal distribution, were represented by median [interquartile range (IQR)], and comparison between the two groups was performed using the Mann–Whitney U test. The categorical variables were expressed as the number of cases (percentage), and the comparison between the two groups was performed using the *χ*^2^ test or Fisher’s exact test. Univariate and multivariate logistic analyses were used to evaluate the related factors affecting DWI lesions and clinical outcome in patients with ICH; *p* < 0.05 was considered statistically significant.

## Results

### Description of all enrolled patients

In our study, a total of 257 patients with ICH were enrolled, and 12 patients were not included in the study because follow-up could not be completed. A total of 245 patients were included in the study for analysis. Of these enrolled patients, 46 (18.78%) had distal DWI lesions after ICH, and 199 (81.22%) had no distal DWI lesions. The average age of 245 patients was 61.98 ± 14.7 years, among whom 158 (64.5%) were male patients. A total of 155 (63.3%) patients had a history of hypertension, and 114 (46.5%) took antihypertensive drugs regularly. The average fasting blood glucose and fasting triglyceride were 5.59 ± 2.63 and 1.70 ± 1.27 mmol/L, respectively. At admission, the average scores of GCS and NIHSS were 14.03 ± 2.16 and 5.77 ± 7.51, respectively. The median time from onset to MRI was 72 (21–192) min, and the median amount of blood loss was 7.19 (2.66–16.65) ml. A total of 121 (49.4%) patients had left ICH, 140 (57.1%) had deep ICH, 51 (20.8%) had intraventricular hemorrhage, 93 (38.0%) had midline shift, and only 11 (4.5%) had subarachnoid hemorrhage. Only 15 (6.1%) patients received surgical treatment, and the remaining 230 (93.9%) patients received conservative treatment. After 3 months, 151 patients (61.6%) had good functional outcomes (mRS 0–2), while 94 (38.4%) had poor functional outcomes (mRS 3–6). The mortality rate was 4.5% (*n* = 11).

### Baseline characteristics of patients with ICH with or without DWI lesions

Baseline characteristics of the comparison between the two groups are presented in Table 1. Compared to the DWI lesions-positive group, patients in the DWI lesions negative group who regularly and consistently took antihypertensive medication (32.6 vs. 49.7%, *p* = 0.048). The C-reactive protein in ICH patients with DWI lesions was higher than that in patients without DWI lesions (14.20 ± 23.72 vs. 7.51 ± 15.01, *p* = 0.017). Other indicators were not statistically significant. Spearman correlation analysis of the risk factors of DWI lesions after ICH is shown in Supplementary Table S3. Antihypertensive treatment was negatively correlated with DWI lesions (r = −0.134, *p* = 0.036), while elevated blood glucose was positively correlated with DWI lesions (r = 0.131, *p* = 0.041).

**Table 1:** Baseline characteristics of patients with ICH with and without DWI lesions.

Characteristics	All ICHs (*n* = 245)	DWI lesions (*n* = 46)	No DWI lesions (*n* = 199)	*p-*value
Age, mean ± SD	61.98 ± 14.7	62.52 ± 13.19	61.85 ± 15.06	0.780
Male, *n* (%)	158 (64.5)	33 (71.7)	125 (62.8)	0.310
Consciousness, *n* (%)				0.817
Awake	189 (77.1)	34 (73.9)	155 (77.9)	
Drowsiness	33 (13.5)	7 (15.2)	26 (13.1)	
Lethargy	12 (4.9)	4 (8.7)	8 (4.0)	
Coma	11 (4.5)	1 (2.2)	10 (5.0)	
Hypertension, *n* (%)	155 (63.3)	24 (52.2)	131 (65.8)	0.060
Diabetes, *n* (%)	23 (9.4)	6 (13.0)	17 (8.5)	0.400
Hyperlipidemia, *n* (%)	7 (2.9)	1 (2.2)	6 (3)	0.750
Atrial fibrillation, *n* (%)	2 (0.8)	0 (0)	2 (1.0)	0.490
Coronary heart disease, *n* (%)	6 (2.4)	0 (0)	6 (3.0)	0.365
History of malignant tumor, *n* (%)	10 (4.1)	4 (8.7)	6 (3.0)	0.096
History of TIA/stroke, *n* (%)	24 (9.8)	3 (6.5)	21 (10.6)	0.440
Smoking, *n* (%)	71 (29)	14 (30.4)	57 (28.6)	0.857
Drinking, *n* (%)	43 (17.6)	10 (21.7)	33 (16.6)	0.519
Antihypertensive therapy, *n* (%)	114 (46.5)	15 (32.6)	99 (49.7)	0.048
Hypoglycemic therapy, *n* (%)	17 (6.9)	5 (10.9)	12 (6.0)	0.329
Statin therapy, *n* (%)	19 (7.8)	4 (8.7)	15 (7.5)	1.000
Treatment (%)				0.743
Conservative	230 (93.9)	44 (95.7)	186 (93.5)	
Surgical	15 (6.1)	2 (4.3)	13 (6.5)	
Time from onset to MRI (h), IQR	72 (21–192)	48 (24–120)	48 (24–96)	0.536
GCS score, mean ± SD	14.03 ± 2.16	13.85 ± 2.23	14.07 ± 2.15	0.530
NIHSS score, mean ± SD	5.77 ± 7.51	5.83 ± 6.745	5.83 ± 7.684	0.789
Admission SBP, (mmHg), mean ± SD	148.11 ± 23.51	149.83 ± 21.71	147.72 ± 23.95	0.585
Admission DBP, (mmHg), mean ± SD	90.91 ± 53.84	86.72 ± 18.99	91.88 ± 59.04	0.559
Fasting blood glucose (mmol/L), mean ± SD	5.79 ± 2.36	6.33 ± 2.77	5.67 ± 2.24	0.088
Total cholesterol (mmol/L), mean ± SD	4.41 ± 1.18	4.41 ± 1.22	4.41 ± 1.17	0.997
Free cholesterol (mmol/L), mean ± SD	1.52 ± 0.49	1.52 ± 0.46	1.52 ± 0.50	0.987
Triglyceride (mmol/L), mean ± SD	1.70 ± 1.27	1.79 ± 2.07	1.67 ± 1.0	0.568
Low-density lipoprotein (mmol/L), mean ± SD	2.63 ± 0.95	2.71 ± 1.05	2.6 ± 0.92	0.540
High-density lipoprotein (mmol/L), mean ± SD	1.17 ± 0.35	1.13 ± 0.36	1.17 ± 0.34	0.454
Hemoglobin (g/L), IQR	144.0 (130.5–155)	140.0 (131.5–152.2)	145.0 (130–156)	0.336
C-reactive protein (g/L), mean ±SD	8.77 ± 17.13	14.20 ± 23.72	7.51 ± 15.01	0.017
D-dimer (mg/L), mean ± SD	1.68 ± 4.00	1.98 ± 3.16	1.63 ± 4.17	0.594
TyG index, mean ± SD	1.37 ± 0.67	1.41 ± 0.63	1.36 ± 0.67	0.660

### Comparison of image characteristics of ICH patients with or without DWI lesions

The comparison of imaging features between the two groups is shown in Table 2. In the group of patients with DWI lesions, the majority (*n* = 21, 45.7%) had lobar bleeding, 20 (43.5%) had deep ICH, 4 (8.7%) had brain stem ICH, while only 1 (2.2%) had bleeding in the cerebellum. However, in the group of patients without DWI lesions, 120 (60.3%) had deep ICH, 45 (22.6%) had lobar bleeding, 18 (9.0%) had brain stem ICH, and 16 (8.0%) had cerebellar ICH. There was statistical significance in the hematoma site (*p* = 0.013). ICH patients were divided into acute and subacute stages according to the time from onset to MRI (26, 27). It is acute within 3 days and subacute within 30 days. There were 180 (73.5%) patients in the acute stage, including 36 (20%) patients with DWI lesions and 144 (80%) patients without DWI lesions. In the subacute stage, 10 (15.4%) patients had DWI lesions, and 55 (84.6%) patients had no DWI lesions. The occurrence of DWI lesions between the acute and subacute phases did not show a significant difference.

**Table 2:** The image features of patients with ICH with and without DWI lesions.

Characteristics	All ICHs (*n* = 245)	DWI lesions (*n* = 46)	No DWI lesions (*n* = 199)	*p-*value
Stage (%)				0.464
Acute stage	180 (73.5)	36 (78.3)	144 (72.4)	
Subacute stage	65 (26.5)	10 (21.7)	55 (27.6)	
Hematoma volume (mL), IQR	7.19 (2.66–16.63)	8.75 (2.97–16.70)	7.07 (2.56–16.93)	0.522
Hematoma side (%)				0.286
Left	121 (49.4)	26 (56.5)	95 (47.7)	
Right	118 (48.2)	18 (39.1)	100 (50.3)	
Middle	6 (2.4)	2 (4.3)	4 (2.0)	
Hematoma site (%)				0.013
Deep ICH	140 (57.1)	20 (43.5)	120 (60.3)	
Lobar ICH	66 (26.9)	21 (45.7)	45 (22.6)	
Brain stem ICH	22 (9)	4 (8.7)	18 (9.0)	
Cerebellar ICH	17 (6.9)	1 (2.2)	16 (8.0)	
Intraventricular hemorrhage, *n* (%)				0.225
Yes	51 (20.8)	13 (28.3)	38 (19.1)	
No	194 (79.2)	33 (71.7)	161 (80.9)	
Subarachnoid hemorrhage (%)				0.695
Yes	11 (4.5)	3 (6.5)	8 (4.0)	
No	234 (95.5)	43 (93.5)	191 (96)	
Midline shift (%)				0.177
Yes	93 (38.0)	13 (28.3)	80 (40.2)	
No	152 (62.0)	33 (71.7)	119 (59.8)	
WMH (%)				0.270
Fazekas level 0	11 (4.5)	1 (2.2)	10 (5.0)	
Fazekas level 1	71 (20.0)	9 (19.6)	62 (31.2)	
Fazekas level 2	66 (26.9)	16 (34.8)	50 (25.1)	
Fazekas level 3	97 (39.6)	20 (43.5)	77 (38.7)	
EPVS (%)				
Basal ganglia-EPVS				0.587
0	8 (3.3)	0 (0.0)	8 (4.0)	
1	16 (6.5)	2 (4.3)	14 (7.0)	
2	43 (17.6)	8 (17.4)	35 (17.6)	
3	67 (27.3)	12 (26.1)	55 (27.6)	
4	111 (45.3)	24 (52.2)	87 (43.7)	
Midbrain-EPVS				1.000
Yes	222 (90.6)	42 (91.3)	180 (90.5)	
No	23 (9.4)	4 (8.7)	19 (9.5)	
Brain atrophy (%)				0.502
Yes	153 (62.4)	15 (32.6)	77 (38.7)	
No	92 (37.6)	31 (67.4)	122 (61.3)	
Lacunar infarction (%)				0.183
Yes	148 (60.4)	32 (69.6)	116 (58.3)	
No	97 (39.6)	14 (30.4)	83 (41.7)	

### Relationship between TyG index and DWI lesions in patients with ICH

The TyG index was calculated based on the fasting blood glucose and triglyceride levels collected. A box-type diagram of the TyG index was created to indicate the presence or absence of DWI lesions, as shown in Supplementary Figure S1. The relationship between the TyG index and DWI lesions in patients with ICH is shown in Supplementary Table S1. The TyG index was divided into four groups according to the interquartile interval (Q1, Q2, Q3, and Q4): TyG < 0.93, 0.93 ≤ TyG < 1.31, 1.31 ≤ TyG < 1.72, and TyG ≥ 1.72. The number of patients in each group was 65 (25.7%), 60 (24.5%), 61 (24.9%), and 61 (24.9%), respectively. There was no significant trend toward higher rates of DWI lesions with increasing TyG index.

Then, all patients with ICH were divided into two groups according to whether they had diabetes or not. There were 23 patients in the diabetes group, including 6 (26.1%) patients with DWI lesions and 17 (73.9%) patients without DWI lesions (Supplementary Table S2). In the diabetes group, the percentage of patients with and without DWI lesions were 1 (16.7%) vs. 2 (11.8%) in Q1, 2 (33.3%) vs. 4 (23.5%) in Q2, 1 (16.7%) vs. 5 (29.4%) in Q3, and 2 (33.3%) vs. 6 (35.3%) in Q4, respectively. There was no significant trend toward higher rates of DWI lesions with increasing TyG index. The same results were seen in the non-diabetic group, the percentage of patients with and without DWI lesions were 9 (22.5%) vs. 51 (28.0%) in Q1, 11 (27.5%) vs. 43 (23.6%) in Q2, 11 (27.5%) vs. 44 (24.2%) in Q3, and 11 (27.5%) vs. 44 (24.2%) in Q4, respectively. Spearman correlation analysis is presented in Supplementary Table S3.

### DWI lesions and clinical outcomes

**Figure 2:**
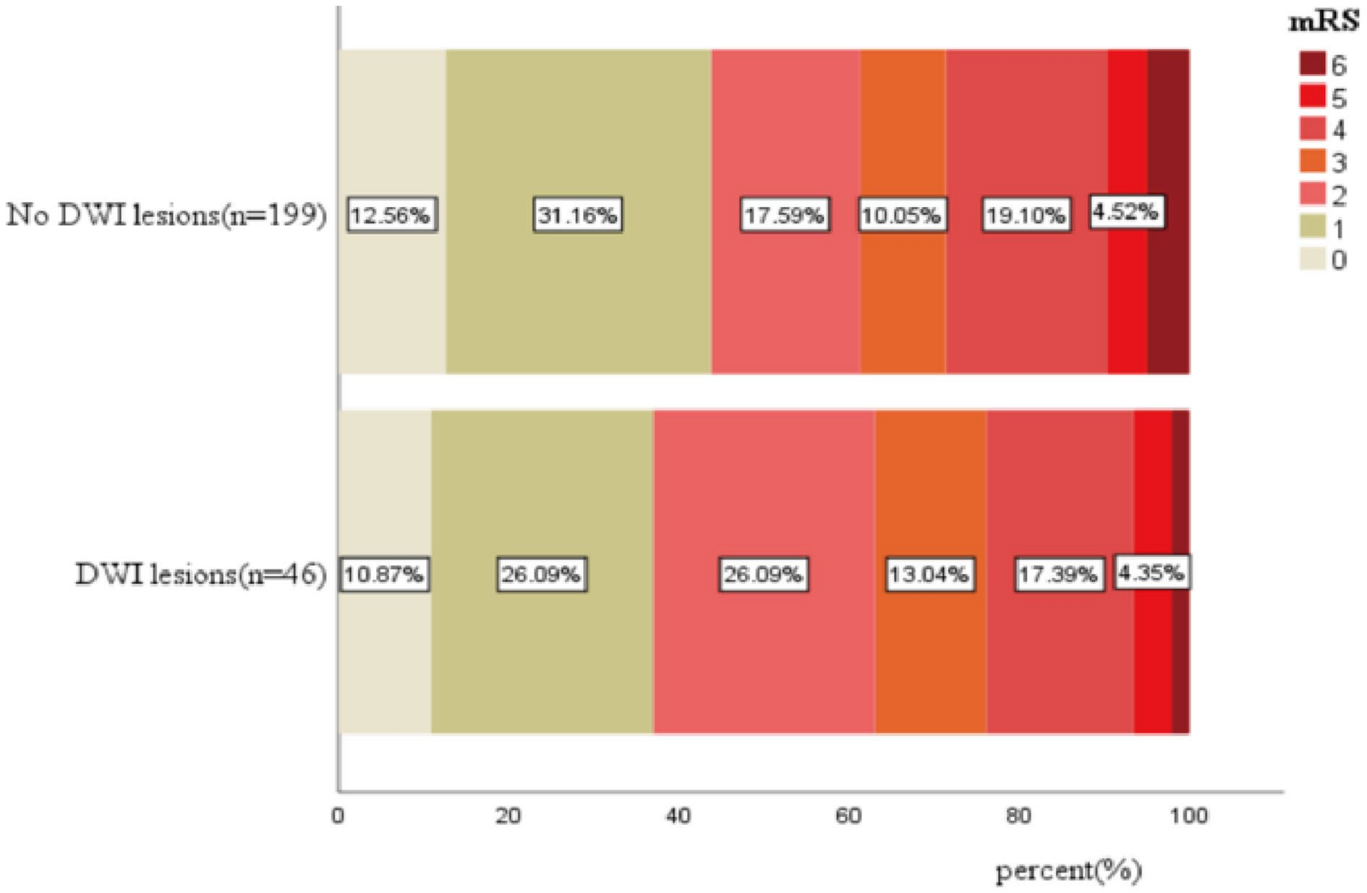
The distribution of mRS score after 3 months in patients with ICH with and without DWI.

The mortality of patients with and without DWI lesions was 1 (2.2%) and 10 (5.0%), which was not significant (*p* = 0.484, Table 3; Figure 2). Similarly, the percentage of patients with poor functional outcomes (mRS 3–6) between the two groups was not significant (37% vs. 38.7%, Table 3; Figure 2).

**Table 3:** The clinical outcome of patients with ICH with and without DWI.

Characteristics	All ICHs (*n* = 245)	DWI lesions (*n* = 46)	No DWI lesions (*n* = 199)	*p-*value
Mortality, *n* (%)	11 (4.5)	1 (2.2)	10 (5.0)	0.484
Functional outcome, *n* (%)				0.868
Good (mRS 0–2)	151 (61.6)	29 (63)	122 (61.3)	
Poor (mRS 3–6)	94 (38.4)	17 (37)	99 (38.7)	
The mRS score at 3 months, *n* (%)				0.837
0	30 (12.2)	5 (10.9)	25 (12.6)	
1	74 (30.2)	12 (26.1)	62 (31.2)	
2	47 (19.2)	12 (26.1)	35 (17.6)	
3	26 (10.6)	6 (13.0)	20 (10.1)	
4	46 (18.8)	8 (17.4)	38 (19.1)	
5	11 (4.5)	2 (4.3)	9 (4.5)	
6	11 (4.5)	1 (2.2)	10 (5.0)	

**Figure 3:**
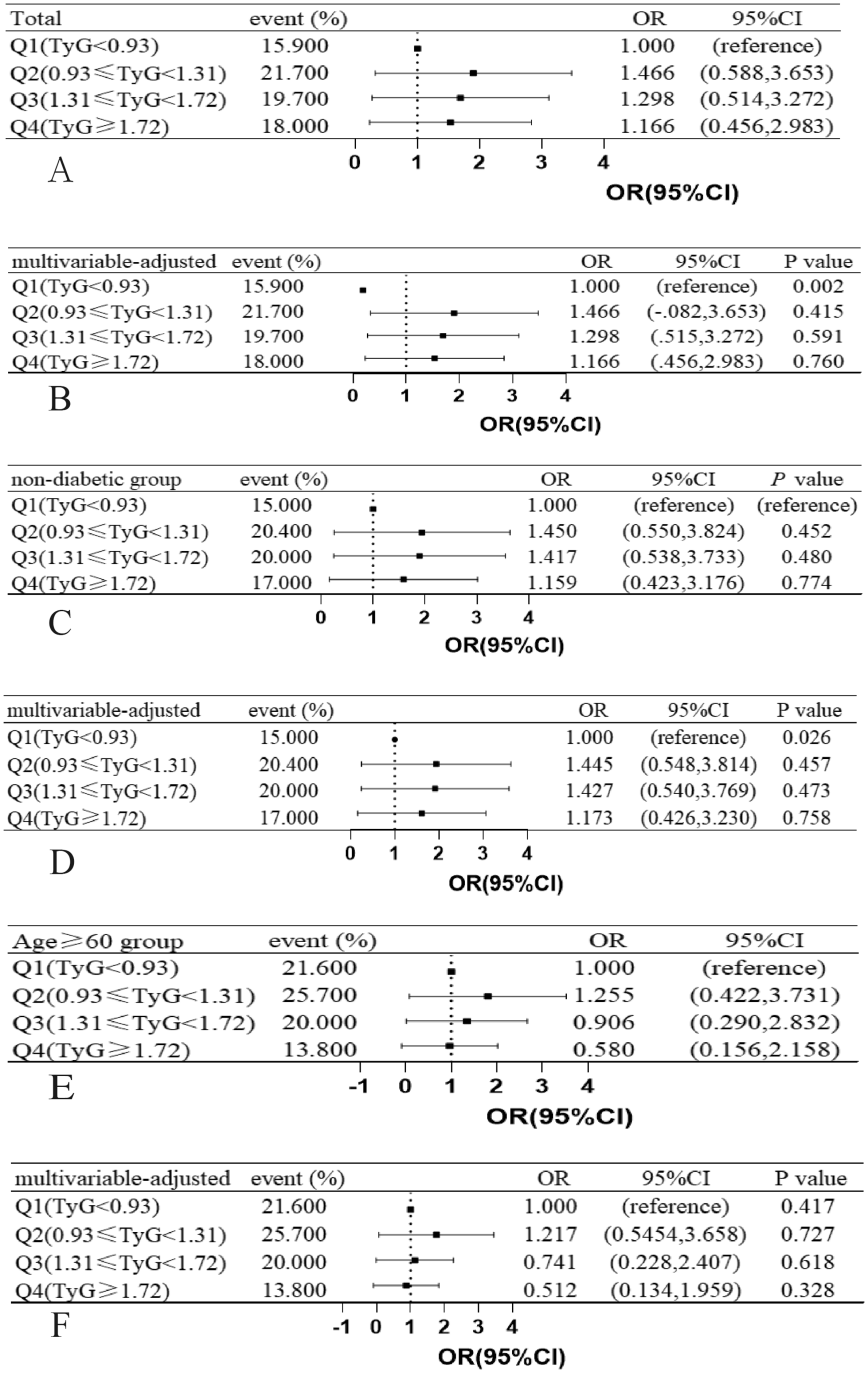
Associations between JyG index and the presence of DWI lesions in **(A,B)** total study patients, **(C,D)** age ≥ 60 years patients, **(E,F)** non-diabetic patients, ORs and 95% CIs of DWI lesions occurrence are shown according to JyG index quartiles in each group. The multivariable-adjusted models were adjusted for age (not for age ≥ 60 years group), history of hypertension, history of diabetes mellitus (not for non-diabetic group). OR indicates odds ratio; CI, confidence interval; Q, quartile; JyG, triglyceride-glucose; DWI, diffusion-weighted imaging.

**Figure 4:**
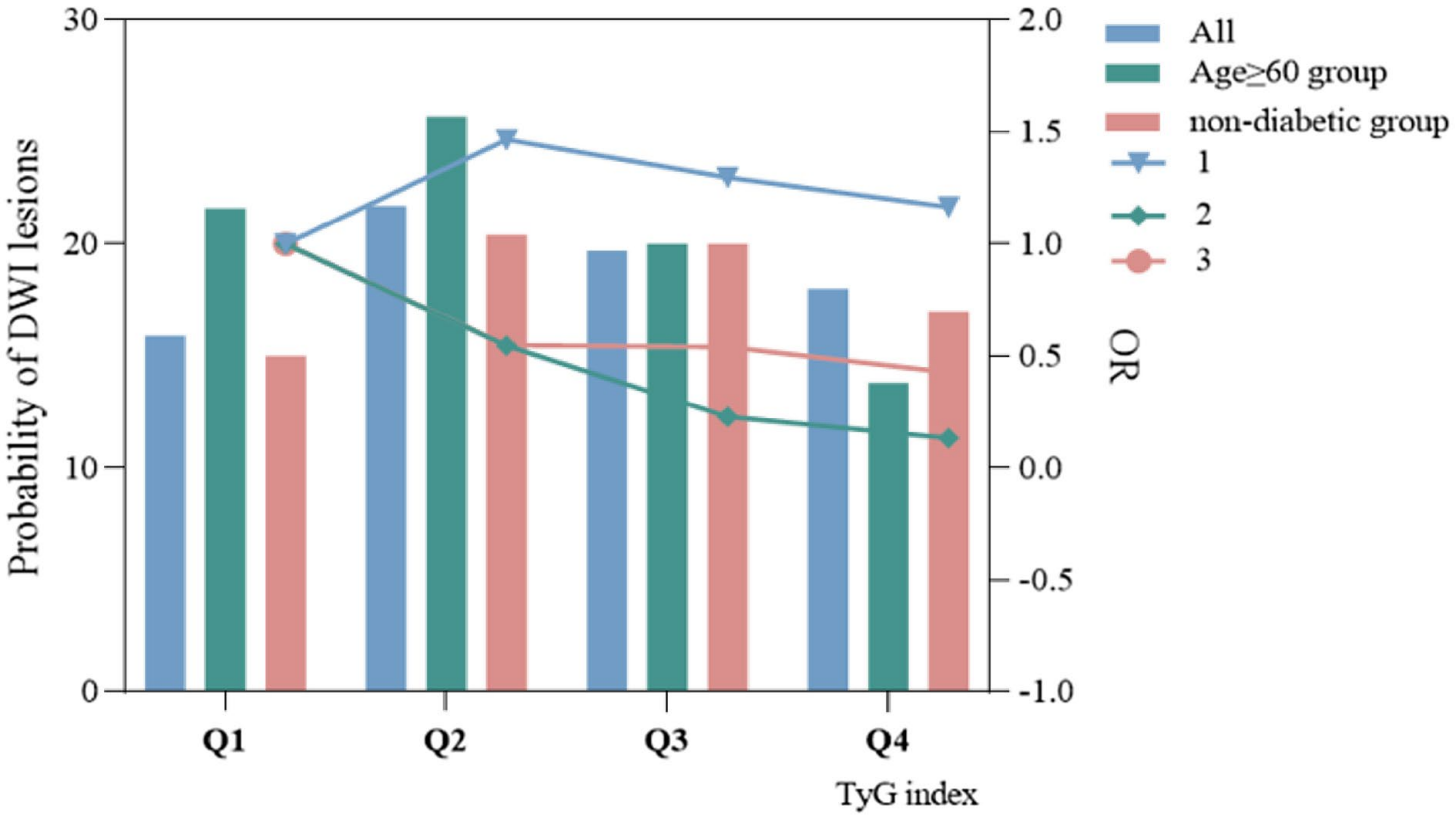
The relationship between TyG index and probability of occurrence of DWI lesions and odds ratio.

In the TyG index quartile of the total patients, a trend was observed where patients with a TyG index in Q2, Q3, and Q4 had a higher risk of DWI lesions compared with patients with a TyG index in Q1 (Figures 3A,B). We did not find a similar pattern in the ≥60-year age group (Figures 3C,D) and the non-diabetic group (Figures 3E,F). However, we observed that in all patients, the non-diabetic group and the ≥60-year age group, the occurrence probability of DWI lesions reached a maximum in the range Q2 of the TyG index, as shown in Figure 4.

### The clinical characteristics between different clinical outcome groups

Comparison of the clinical characteristics and imaging data between ICH patients with favorable functional outcome (*n* = 152) and those with unfavorable functional outcome (*n* = 95) at 90 days (Tables 4, 5). Long-term non-smokers were more likely to have favorable functional outcomes at 90 days after onset (66.2% vs. 33.8%, *p* = 0.043). The overall consciousness scores showed significant differences between the two groups (*p* < 0.001). The patients in the unfavorable functional outcome group were associated with a higher rate of midline shift (58.1% vs. 41.9%, *p* < 0.001), surgical treatment (80.0% vs. 20.0%, *p* = 0.001), and intraventricular hemorrhage (68.6% vs. 31.4%, *p* < 0.001). Compared with patients with poor outcome, patients with good clinical outcome had higher GCS score (14.7 ± 1.12 vs. 12.95 ± 2.88, *p* < 0.001), lower NIHSS score (2.25 ± 3.89 vs. 10.94 ± 8.92, *p* < 0.001), lower systolic blood pressure (SBP) (145.05 ± 34.15 vs. 153.04 ± 21.68, *p* = 0.009), lower fasting blood glucose level (5.53 ± 2.09 vs. 6.23 ± 2.69, *p* = 0.021), and C-reactive protein levels (6.30 ± 13.54 vs. 0.12.72 ± 21.17, *p* = 0.004). The hematoma volume in the group with good clinical outcome was smaller than that in the group with poor clinical outcome [4.46 (1.59–11.45) vs. 12.44 (6.69–26.47), *p* < 0.001].

**Table 4:** Comparison of characteristics of patients with ICH with different clinical outcomes.

Characteristics	All ICHs (*n* = 245)	Good outcome (*n* = 151)	Poor outcome (*n* = 94)	*p-*value
Age, mean ±SD	61.98 ± 14.7	59.15 ± 14.62	66.51 ± 13.74	<0.001
Male, *n* (%)	158 (64.5)	96 (63.6)	62 (66.0)	0.784
Consciousness, *n* (%)				<0.001
Awake	189 (77.1)	136 (90.1)	53 (56.4)	
Drowsiness	33 (13.5)	12 (7.9)	21 (22.3)	
Lethargy	12 (4.9)	0 (0)	12 (12.8)	
Coma	11 (4.5)	3 (2.0)	8 (8.5)	
Hypertension, *n* (%)	155 (63.3)	95 (62.9)	60 (63.8)	0.893
Diabetes, *n* (%)	23 (9.4)	14 (9.3)	9 (9.6)	1.000
Hyperlipidemia, *n* (%)	7 (2.9)	3 (2.0)	4 (4.3)	0.433
Atrial fibrillation, *n* (%)	2 (0.8)	2 (1.3)	0 (0)	0.525
Coronary heart disease, *n* (%)	6 (2.4)	5 (3.3)	1 (1.1)	0.411
History of malignant tumor, *n* (%)	10 (4.1)	5 (3.3)	5 (5.3)	0.513
History of TIA/stroke, *n* (%)	24 (9.8)	17 (11.3)	7 (7.4)	0.383
Smoking, *n* (%)	71 (29)	51 (33.8)	20 (21.3)	0.043
Drinking, *n* (%)	43 (17.6)	32 (21.2)	11 (11.7)	0.083
Antihypertensive therapy, *n* (%)	114 (46.5)	68 (45.0)	46 (48.9)	0.599
Hypoglycemic therapy, *n* (%)	17 (6.9)	19 (6.6)	7 (7.4)	1.000
Statin therapy, *n* (%)	19 (7.8)	9 (6.0)	10 (10.6)	0.221
Treatment (%)				0.001
Conservative	230 (93.9)	148 (98.0)	82 (87.2)	
Surgical	15 (6.1)	3 (2.0)	12 (12.8)	
GCS score, mean ± SD	14.03 ± 2.16	14.7 ± 1.12	12.95 ± 2.88	<0.001
NIHSS score, mean ± SD	5.77 ± 7.51	2.55 ± 3.89	10.94 ± 8.92	<0.001
Admission SBP, (mmHg), mean ± SD	148.11 ± 23.51	145.05 ± 24.15	153.04 ± 21.68	0.009
Admission DBP, (mmHg), mean ± SD	90.91 ± 53.84	92.03 ± 67.49	89.12 ± 15.89	0.682
Fasting blood glucose (mmol/L), mean ± SD	5.79 ± 2.36	5.52 ± 2.09	6.23 ± 2.69	0.021
Total cholesterol (mmol/L), mean ± SD	4.41 ± 1.18	4.48 ± 1.13	4.30 ± 1.25	0.254
Free cholesterol (mmol/L), mean ± SD	1.52 ± 0.49	1.52 ± 0.42	1.52 ± 0.59	0.905
Triglyceride (mmol/L), mean ± SD	1.70 ± 1.27	1.71 ± 1.02	1.57 ± 0.91	0.277
Low-density lipoprotein (mmol/L), mean ± SD	2.63 ± 0.95	2.72 ± 0.98	2.50 ± 0.88	0.090
High-density lipoprotein (mmol/L), mean ± SD	1.17 ± 0.35	1.15 ± 0.34	1.18 ± 0.36	0.438
Hemoglobin (g/L), IQR	144.0 (130.5–155)	146.0 (134.0–156.0)	141.5 (126.75–153.25)	0.129
C-reactive protein (g/L), mean ± SD	8.77 ± 17.13	6.30 ± 13.54	12.72 ± 21.17	0.004
TyG index, mean ± SD	1.37 ± 0.67	1.37 ± 0.64	1.38 ± 0.711	0.859

**Table 5:** Comparison of imaging features of patients with ICH with different clinical outcomes.

Characteristics	All ICHs (*n* = 245)	Good outcome (*n* = 151)	Poor outcome (*n* = 94)	*p-*value
Hematoma volume (mL), IQR	7.19 (2.66–16.63)	4.46 (1.59–11.45)	12.44 (6.69–26.47)	<0.001
Hematoma side (%)				0.961
Left	121 (49.4)	74 (49.1)	47 (50.0)	
Right	118 (48.2)	73 (48.3)	45 (47.9)	
Middle	6 (2.4)	4 (2.6)	4 (2.1)	
Hematoma site (%)				0.098
Deep ICH	140 (57.1)	79 (52.3)	61 (64.9)	
Lobar ICH	66 (26.9)	42 (27.8)	24 (25.5)	
Brain stem ICH	22 (9)	18 (11.9)	4 (4.3)	
Cerebellar ICH	17 (6.9)	12 (8.0)	5 (5.3)	
Intraventricular hemorrhage, *n* (%)				<0.001
Yes	51 (20.8)	16 (10.6)	35 (37.2)	
No	194 (79.2)	135 (89.4)	59 (62.8)	
Subarachnoid hemorrhage (%)				0.111
Yes	11 (4.5)	4 (2.6)	7 (7.4)	
No	234 (95.5)	147 (97.4)	87 (92.6)	
Midline shift (%)				<0.001
Yes	93 (38.0)	39 (25.8)	54 (57.4)	
No	152 (62.0)	112 (74.2)	40 (42.6)	
DWI lesions (%)				0.868
Yes	46 (18.8)	29 (19.2)	17 (18.1)	
No	199 (81.2)	122 (80.8)	77 (81.9)	
WMH (%)				0.630
Fazekas level 0	11 (4.5)	7 (4.6)	4 (4.3)	
Fazekas level 1	71 (20.0)	48 (31.8)	23 (24.5)	
Fazekas level 2	66 (26.9)	40 (26.5)	26 (27.7)	
Fazekas level 3	97 (39.6)	56 (37.1)	41 (43.6)	
EPVS (%)				
Basal ganglia-EPVS				0.832
0	8 (3.3)	5 (3.3)	3 (3.3)	
1	16 (6.5)	12 (7.9)	16 (6.5)	
2	43 (17.6)	27 (17.9)	43 (17.6)	
3	67 (27.3)	41 (27.2)	67 (27.3)	
4	111 (45.3)	66 (43.7)	111 (45.3)	
Midbrain -EPVS				1.000
Yes	222 (90.6)	137 (90.7)	85 (90.4)	
No	23 (9.4)	14 (9.3)	9 (9.6)	
Brain atrophy (%)				0.030
Yes	153 (62.4)	86 (57.0)	67 (71.3)	
No	92 (37.6)	65 (43.0)	27 (28.7)	
Lacunar infarction (%)				0.422
Yes	148 (60.4)	88 (58.3)	60 (63.8)	
No	97 (39.6)	63 (41.7)	34 (36.2)	

### Multivariable logistic regression analysis of DWI lesion occurrence

**Figure 5:**
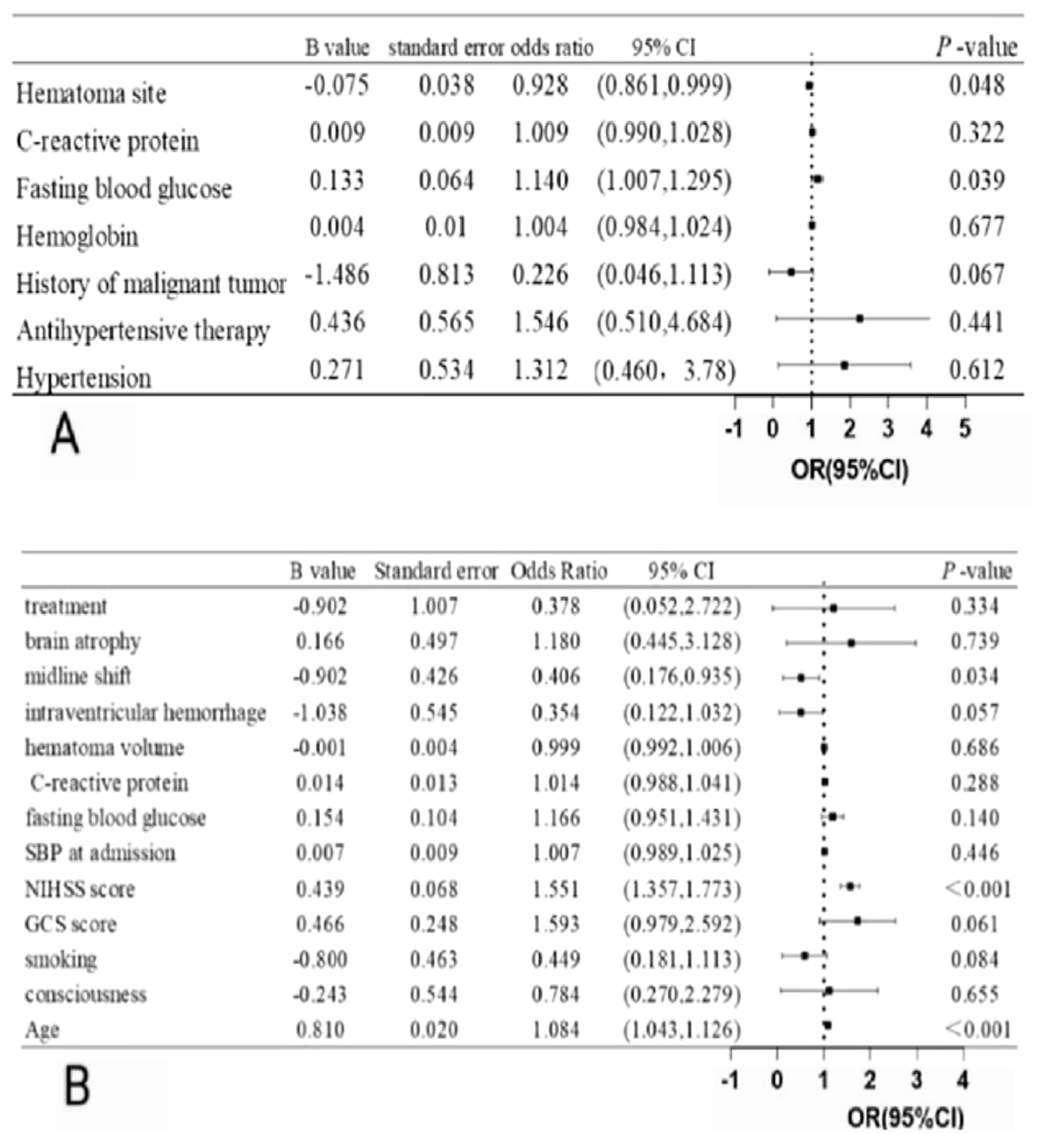
**(A)** Multivariate logistic analysis of DWI lesions after ICH **(B)** Multivariate logistic analysis of clinical outcome after ICH.

Univariate analysis identified the following factors as being related to the occurrence of DWI lesions: history of hypertension, use of antihypertensive medication, history of malignancy, hemoglobin concentration, fasting blood glucose, C-reactive protein, and hematoma site. The multivariate logistic regression showed that high fasting glucose [odds ratio (OR) = 1.140, 95% confidence interval (CI): 1.007–1.295, *p* = 0.039] and hematoma site (OR = 0.928, 95% CI: 0.861 = 0.999, *p* = 0.048) were significant predictors of DWI lesions (Figure 5A).

### Multivariable logistic regression analysis of 90-day clinical outcomes

Univariate analysis determined the following elements as being related to a 3-month poor functional outcome: age, consciousness, smoking, GCS score, NIHSS score, SBP at admission, fasting blood glucose, C-reactive protein, hematoma volume, intraventricular hemorrhage, midline shift, brain atrophy, and treatment. The multivariate logistic regression showed that age (OR = 1.084, 95% CI: 1.043–1.126, *p* < 0.001), NIHSS score (OR = 1.551, 95% CI: 1.357–1.733, *p* < 0.001), and midline shift (OR = 0.406, 95%CI: 0.176–0.935, *p* = 0.034) were independent predictor of poor functional outcome at 90 days (Figure 5B).

## Discussion

Our study compared the clinical baseline data, image features, treatment, and clinical outcome of ICH patients with and without DWI lesions. The results showed that compared with the negative group of DWI lesion, the positive group of DWI lesion was higher in patients with elevated fasting blood glucose and a higher rate of lobar hemorrhage. However, there was no significant difference in clinical outcomes between the two groups. Nevertheless, age, NIHSS score, and midline shift were the significant predictors of poor clinical outcome for patients with ICH.

Currently, the mechanism underlying DWI lesion formation following ICH remains unclear. Some studies had suggested that it was related to the rapid decrease in blood pressure in the acute phase (28), and changes in mean arterial pressure before MRI examination were associated with the presence of DWI lesion (9, 29). It is widely acknowledged that early antihypertensive treatment was necessary for patients with ICH. However, for patients with extremely high blood pressure within the first 24 h, which may lead to suboptimal cerebral perfusion and is related to the occurrence of DWI lesions after ICH. However, due to the lack of continuous blood pressure monitoring for our patients, we did not analyze this aspect in our study. Another study indicated that the presence of multiple DWI lesions after ICH may indicate the existence of cardiac emboli (21). However, there is no evidence linking DWI lesions and a history of atrial fibrillation.

Other studies have shown that DWI lesions are associated with the cascade of acute pro-thrombosis and pro-inflammation triggered by ICH (30–32). Oxidative stress and inflammation after ICH might result in thrombosis and DWI lesions (33). IR was associated with neuroinflammation and was considered one of the primary culprits in neurodegenerative processes. IR was a systemic metabolic abnormality with a pro-inflammatory effect, and it had been proven to be closely associated with metabolic disorders, including blood glucose, blood lipids, and blood pressure (34). IR disrupted insulin signaling at the cellular level in the intima by activating inflammation-related genes (10, 35), leading to varying degrees of oxidation, chronic inflammation, and endothelial dysfunction (36). This impairment also hindered vascular remodeling and growth, ultimately contributing to the development of cerebrovascular disease (37). IR increased blood coagulation capacity by promoting platelet activation, adhesion, and aggregation, which led to hemodynamic changes and increased the chance of thrombosis (38) and correspondingly triggered cerebrovascular lesions. The TyG index was used as an alternative marker for insulin resistance. IR was a disease characterized by impaired tissue response to insulin stimulation. It is the main feature of metabolic diseases and can lead to disorders of glycolipid metabolism. A large number of studies confirmed that it was related to various diseases, such as atherosclerosis, carotid plaque formation and rupture, hyperglycemia, dyslipidemia, coronary artery disease, pulse disease, stroke, etc. (9, 39, 40), and played a very important role in cardiovascular and cerebrovascular diseases.

A meta-analysis found that elevated TyG index was a risk factor for ischemic stroke, while the correlation between the two was non-linear (13). Another study also had a similar finding that individuals with a high TyG index were more likely to develop cerebrovascular disease (37). Therefore, we hypothesized that IR was associated with the formation of DWI lesions in patients with ICH. We attempted to find the relationship between the TyG index and DWI lesions. A previous study has shown that there was an independent correlation between the HOMA-IR index and DWI lesions in patients with ICH (34). In our study, we found that the incidence of DWI lesions in each patient group, specifically the non-diabetic group and the age group above 60 years in the Q2 interval, showed a relatively higher trend; however, the difference was not statistically significant. The reasons may be as follows: the sample size of this study was relatively small, which might lead to sample size bias, and a small number of DWI lesions had a significant impact on the statistical incidence results. In the future, we expect to expand the sample size and conduct multi-center studies to try to find a clear correlation between them. If we can establish the relationship between them, the TyG index can be controlled within a certain range clinically, thereby reducing the probability of DWI lesions in patients with ICH and improving the clinical outcome of patients with ICH.

A previous study has shown that DWI lesions were most likely to occur within 48 to 72 h after ICH (41). In our study, the median time for DWI lesions to occur was 48 h, which was consistent with the results of previous studies. Although we considered that scanning time might affect the detection of DWI lesions when designing the study, we divided ICH patients into two stages—the acute stage and the subacute—for analysis according to different time periods. However, no statistically significant differences were observed. The DWI lesions may occur before and after the scanning. However, due to factors such as the cost of MRI examinations, it is impossible to conduct repeated scanning examinations for each patient. Therefore, our study only recorded the initial MRI results of patients with ICH.

In our study, we found that fasting glucose was an independent risk factor for the development of DWI lesions, consistent with previous studies (21). Hyperglycemia during ICH may be categorized in the following types: (1) Preexisting hyperglycemia aggravated by ICH-related stress. (2) Patients with normal blood glucose levels were briefly elevated and increased with the onset of ICH, which was consistent with previously proposed hypotheses (42, 43) that hyperglycemia may be a transient neuroendocrine change that occurred during intracerebral injury, which was a cause for alarm (44). In the context of ICH, tension and stress, as well as cortisol and catecholamine hormones, led to spasms of microvessels, resulting in hyperglycemia and small infarctions.

In addition, age, NIHSS score, and midline shift were identified as independent risk factors affecting clinical outcomes of patients with ICH, which was consistent with previous studies (44). All these indicators were used to assess clinical severity, and clinical severity was found to be associated with prognosis. Previous studies indicated that patients with DWI lesions after ICH had poorer clinical outcomes than patients without DWI lesions (7, 40). However, our study showed that the frequency of death or disability within 90 days in patients with DWI lesions was similar to that in patients without DWI lesions. Apart from the influence of sample size, another possible reason was that our study’s follow-up method was conducted through phone calls. The information obtained through phone follow-up was affected by the comprehension abilities of patients and their families, as well as subjective factors.

### Limitations

Our study had several limitations. First, this study was a retrospective study, which inherently introduced biases that can impact the study’s results. Second, the measurement of brain volume was based on the initial computed tomography image, which may not account for patients with enlarged hematomas. Third, as a single-center study, the sample size was relatively small, which may have led to potential sample size bias. To address this issue, future research will aim to conduct multi-center studies and increase the sample size. The data we collected included fasting blood glucose levels and fasting triglyceride levels. Considering the time lag between the occurrence of intracerebral hemorrhage and the blood sampling time, fasting blood glucose can be affected by the stress response. The blood glucose levels of patients whose blood was drawn closer to the time of onset remained in a stress-induced elevated state. Therefore, the shorter the interval between fasting blood glucose measurement and the onset of ICH, the lower the blood glucose error. The fasting triglycerides were affected by a history of hyperlipidemia and recent dietary intake. To avoid the influence of fasting triglycerides on the results, multiple measurements are needed.

## Conclusion

In this study, no significant correlation was found between the TyG index and distal DWI lesions. Elevated high fasting glucose levels and hematoma site were independent predictors of DWI lesions after ICH. The age, NIHSS score, and midline shift were independent predictors of poor functional outcome at 90 days. Large-scale studies are still needed to find a link between the TyG index and DWI lesions in ICH patients. Further research is warranted to understand the mechanism underlying these lesions.

## Data Availability

The raw data supporting the conclusions of this article will be made available by the authors, without undue reservation.
