# Supplementary material for: The relationship between remote diffusion-weighted imaging lesions and the triglyceride-glucose index and clinical outcomes in patients with intracerebral hemorrhage
Source: Front Neurol. 2025 Jul 21;16:1562361. doi: 10.3389/fneur.2025.1562361 (PMC12318738; doi:10.3389/fneur.2025.1562361)

Table S1: Comparison of different levels of TyG index in patients with ICH with and without DWI lesions.

| **Characteristics** | **All ICHs**  **(n=245)** | **DWI lesions**  **(n=46)** | **No DWI lesions (n=199)** | ***p-value*** |
| --- | --- | --- | --- | --- |
| **TyG index** |  |  |  | 0.870 |
| TyG<0.93 | 65 (25.7) | 10 (21.7) | 53 (26.6) |  |
| 0.93≤TyG<1.31 | 60 (24.5) | 13 (28.3) | 47 (23.6) |  |
| 1.31≤TyG<1.72 | 61 (24.9) | 12 (26.1) | 49 (24.6) |  |
| TyG≥1.72 | 61 (24.9) | 11 (23.9) | 50 (25.1) |  |

Table S2: Comparison of different levels of TyG index in different groups of ICH patients with and without DWI lesions.

| **Characteristics** | **Diabetic group** | | | | **No-Diabetic group** | | | |
| --- | --- | --- | --- | --- | --- | --- | --- | --- |
|  | **All ICHs**  **(n=23)** | **DWI lesions**  **(n=6)** | **No DWI lesions**  **(n=17)** | ***p-value*** | **All ICHs**  **(n=222)** | **DWI lesions**  **(n=40)** | **No DWI lesions**  **(n=182)** | ***p-value*** |
| **TyG index** |  |  |  | 0.910 |  |  |  | 0.858 |
| TyG<0.93 | 3 (13.0%) | 1 (16.7%) | 2 (11.8%) |  | 60 (27.0%) | 9 (22.5%) | 51 (28.0%) |  |
| 0.93≤TyG<1.31 | 6 (26.1%) | 2 (33.3%) | 4 (23.5%) |  | 54 (24.3%) | 11 (27.5%) | 43 (23.6%) |  |
| 1.31≤TyG<1.72 | 6 (26.1%) | 1 (16.7%) | 5 (29.4%) |  | 55 (24.8%) | 11 (27.5%) | 44 (24.2%) |  |
| TyG≥1.72 | 8 (34.8%) | 2 (33.3%) | 6 (35.3%) |  | 53 (23.9%) | 11 (27.5%) | 44 (24.2%) |  |

Table S3: Superman correlation analysis of risk factors of DWI lesions after ICH.

| **Variable** | ***r_s_*** | ***P-value*** |
| --- | --- | --- |
| Antihypertensive therapy | -0.134 | 0.036 |
| Fasting blood glucose | 0.131 | 0.041 |

Figure S1: Comparison of TyG index with DWI lesion negative group and positive group.


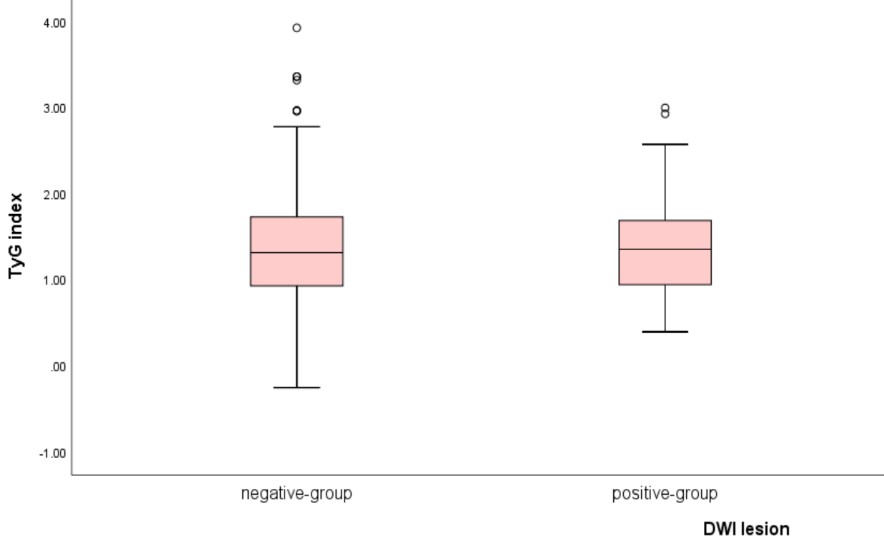

Supplement: Supplementary file 1 [file Table_1.docx]
